# Government Commission for Investigating Yellow Fever Inoculation

**Published:** 1886-02

**Authors:** 


### GOVERNMENT COMMISSION FOR INVESTIGATING YELLOW FEVER INOCULATION.

---

A leader in the *Medical Record* of January 9th, upon "The Question of Government Patronage for Medical Investigation," indicates the great importance of affording wider opportunity for profound study of physiology and the workings of disease. Two great requisites are needful, time and money, and it is seldom that the medical scientist has both at his command. Americans are peculiarly adapted to become investigators in physiology and the art of healing, and if proper encouragement were given them, the world would look to them, as it now does to France and Germany, for the elucidation of wonderful theories relating to disease and human welfare. Europeans have made strides when we have crept because government patronage has been extended to the anatomist and physiologist.

In order to combat disease correct theories are necessary, based on absolute fact as far as possible. The miscroscope and test-tube have revolutionized the science of medicine, and since the welfare of the people—nay, their very lives—depend on the working out of those theories, every means and every encouragement should be given to those who attempt it. To protect the interests of its people is surely the highest duty of any government, monarchical or republican.

With this abstract of the position of our able contemporary, our readers are prepared to appreciate a brief study of the process of inoculation against yellow fever by Dr. Domingos Freire, under the auspices of the Imperial Government at the city of Rio de Janeiro in Brazil, and their attention is called to the facts of the case.

We have at various times presented extracts in our columns touching this prophylactic measure, and the following will be found in the August number at page 383 of the JOURNAL for 1884.

The micrococcus of yellow fever has been discussed by Dr. Domingos Freire, of Rio de Janeiro. He claims to have discovered this in the vomit, the blood and the different secretions of yellow fever patients, and calls it micrococcus xanthogenus. This organism can be found in abundance in the earth of a cemetery at Rio de Janeiro, and even in the soil of the whole city. The Doctor has succeeded in inoculating some animals with yellow fever, and by cultivating the virus to the fifth and sixth generation has attenuated it so that it becomes a vaccine.

It is known that Dr. Freire's investigations were in progress since the year 1880, and we are informed by Dr. Gaston, who spent many years in Brazil, that, prior to his leaving that country in 1883, these experiments of Dr. Domingos Freire had secured the favorable notice of prominent members of the Medical Faculty at Rio de Janeiro. Being thoroughly convinced by what he knew of the scientific attainments of this eminent biologist and microscopist, and by the reports and communications received from competent observers of his proceedings during the past two years, Dr. Gaston felt called upon to bring this important subject to the attention of the Medical Department, and subsequently to the consideration of the President of the United States, with a view to secure authoritative action for the confirmation or rejection of the claims of inoculation as a prophylactic against yellow fever.

The *Rio News*, in the English language, with its editorial management in the hands of a literary gentleman from the United States, has endorsed the trustworthiness of the reported results of Dr. Freire's investigations, and extracts from it have appeared in the *Medical and Surgical Reporter*, as well as in the Atlanta *Evening Journal*, five or six months ago.

A correspondence, published in the New Orleans papers recently, gives a letter of Dr. Domingos Freire to Dr. Gaston, in which it is stated that "the symptoms following inoculation are those which characterize the prodomata of the disease, frontal and intro-orbicular pain in the head, lying down, slight febrile disturbance, sometimes nausea and even vomiting, pain in the epigastric region, with weakness, etc. The phenomena last but two or three days and require no medication."

A strong appeal was made to Dr. Joseph Holt, and in accordance with the suggestions of Dr. Gaston, a proposition was laid before the American Public Health Association, at its recent meeting in the city of Washington, urging the favorable consideration of this matter, and it was recommended by that body to the attention of Congress. Bills embodying the appointment of three competent physicians as a commission for the investigation of the merits of inoculation as a preventive of yellow fever have been filed in both houses of Congress. The views of Dr. Sternberg, a most accurate observer of microscopic phenomena, encourage the prosecution of this inquiry; and in the *Times-Democrat*, of December 31st, it is stated, as we infer in regard to him, as follows:

We have just seen a letter to Dr. Holt from one of the ablest scientists of this country, wherein it is said that the researches in South America and Mexico are "entitled to consideration, and in view of the vast practical importance of the question involved, I think you are quite justified in urging upon Congress the propriety of causing a full investigation to be made by competent persons." While it may be premature to say anything touching the elements which should enter into the composition of this commission, it is evident that the three members should combine the requisites for scientific discrimination in bacteriology, for experimental observation of the types of yellow fever, and for profitable association with the medical men of the regions visited and examined by the commission. We have no doubt that the authorities who may be entrusted with laying this selection before the President will, at the proper time, discern these qualifications in three persons who appear to us pre-eminently fitted for undertaking this arduous service. If the present onerous duties of the professional gentlemen to whom we refer should not preclude their acceptance of such positions, the President would most assuredly have a guarantee for the faithful and efficient performance of this task by entrusting them with this important investigation in Rio de Janeiro, and such other points as may be visited.

There has been a precedent established by our government in sending a distinguished member of the medical profession to study the means of arresting cholera, should it invade this country, and the far-seeing policy of our National Assembly cannot fail to recognize the pressing obligation to adopt this measure for deciding upon the efficacy of so simple a measure as inoculation for stripping the scourge of yellow fever of its terrors in our Southern ports. We await the final action of Congress on this bill with confidence in the wisdom of our legislators, and rely upon the discretion of the President to select the men in the profession with the highest qualifications for this work in the department of protection and best fitted to secure a satisfactory result in this undertaking for the benefit of our people.

---

EXTRAORDINARY FECUNDITY.—Dr. H. L. Battle, of Wadley, Ga., sends us the following remarkable case:

"Anna T., a stout, healthy negress, married, age 24, the mother of four children, was delivered January 1, 1885, of a stout, healthy male child. It lived until March 11, 1885, at which date it died of acute bronchial catarrh. October 1, 1885, I delivered her of another fine, healthy boy, who is still alive at the date of this writing, January 1, 1886. This last child cried lustily at its birth, had a full head of hair, toe and finger nails well developed and could not have been less than eight months. Just nine months between the children. How is that for fast?"

